# The Relationship Between Social Support and Ostracism Among College Students: The Mediating Role of Social Connectedness and the Moderating Role of Flourishing

**DOI:** 10.3390/bs15091198

**Published:** 2025-09-03

**Authors:** Dongying Liu, Zheng Wang, Yingjin Wang, Wenbo Yu, Hongjuan Wang

**Affiliations:** 1 School of Literature, Heilongjiang University, Harbin 150080, Chinawyj247556834@163.com (Y.W.); 2 School of Public Management, Guangzhou Nanfang College, Guangzhou 510970, China; 3 School of Chinese Language and Culture, Nanjing Normal University, Nanjing 210097, China

**Keywords:** social support, ostracism, social connectedness, flourishing

## Abstract

While social support is recognized as a protective factor against ostracism, its underlying psychological mechanisms remain unclear. This study empirically examined the relationship between social support and ostracism, and further tested the mediating role of social connectedness and the moderating effect of flourishing. A total of 723 college students (40.0% male) with a mean age of 19.39 years (*SD* = 0.91) were recruited in this study using the Multidimensional Scale of Perceived Social Support, the Ostracism Experience Scale for Adolescents, the Social Connectedness Scale, and the Flourishing Scale. The findings were as follows: (1) social support was significantly and negatively associated with ostracism; (2) social connectedness mediated the relationship between social support and ostracism; (3) flourishing moderated the relationship between social support and social connectedness; specifically, individuals with higher levels of flourishing may establish and maintain social connectedness using social support more effectively. This study contributes to the existing theoretical framework about the influencing factors of college students’ mental health but also provides new perspectives on mental health education and intervention strategies at school.

## 1. Introduction

Given the growing social concern for mental health, ostracism among college students has emerged as a critical issue. However, previous research on ostracism in college students has mainly focused on its negative consequences, such as mental health problems (Li et al., 2021; Niu et al., 2023). Nevertheless, research on mitigating the adverse effects of ostracism remains limited. Especially, the role of social support in this process, including its underlying mechanisms and boundary conditions, lacks systematic investigation. To address this gap, this study constructs a moderated mediation model proposing that social support reduces ostracism by enhancing college students’ social connectedness, with flourishing potentially moderating the relationship between social support and social connectedness. This model addresses theoretical gaps in mechanistic pathways and boundary conditions while elucidating how and under what circumstances social support influences ostracism, thereby informing targeted intervention strategies.

### 1.1. Social Support and Ostracism

Social support refers to an individual’s network of psychological and material resources designed to enhance their capacity to cope with adversity, which is fundamentally associated with the functioning and quality of social relationships (Cohen, 2004; Schwarzer & Knoll, 2007). Research has shown that social support is regarded as a significant protective factor with a positive relationship with psychological well-being, which can mitigate the adverse effects of external stressors on individuals (Gençöz & Özlale, 2004; Lavasani et al., 2011). In contrast to the adaptive functions of social support, ostracism represents a negative interpersonal experience characterized by intentional exclusion or ignorance within social contexts (Williams, 2007a). Empirical evidence indicates that ostracism threatens the satisfaction of four fundamental human needs: belongingness, self-esteem, control, and meaningful existence (Williams, 2002). When these core psychological needs are compromised, ostracism can adversely affect affective states, cognitive processing, and mental health (Williams, 2007b, 2009). Consequently, ostracism is recognized as a critical social risk factor, with significant differential mechanisms in contrast to the protective effects of social support. Recent studies have examined how social support acts as a buffering mechanism to mitigate the adverse impacts of ostracism (Morese et al., 2019; Scott et al., 2014; Teng & Chen, 2012). In addition, a meta-analysis study also found that a good social support network could enhance individuals’ mental health and help them maintain a positive mindset in the face of stress and challenges (Harandi et al., 2017). For college students, social support is more important (Hu et al., 2022). They are renavigating a transitional period marked by increased autonomy and shifting social roles. Adequate social support not only helps college students to cope with academic, life, and emotional challenges positively and effectively, but may also reduce the negative impact of ostracism. Therefore, based on previous studies, this study explores the relationship between social support and ostracism among college students, and proposes Hypothesis 1 (H1): Social support is significantly and negatively associated with ostracism among college students.

### 1.2. The Mediating Role of Social Connectedness

Social connectedness refers to a measurement representing an individual’s sense of belonging and mutual interaction with others, serving as a critical influencing factor of psychological well-being (Cornwell & Waite, 2009; Wickramaratne et al., 2022). Social connectedness may be a potential mediating mechanism between social support and ostracism. On the one hand, there is a strong link between social support and social connectedness. Social support emphasizes the accessibility of external resources, primarily manifested through assistance and encouragement from family, friends, or community members (Lu & Chen, 1996; Zimet et al., 1988). Such support not only facilitates individual development but also fosters supportive interactions and a stronger sense of belonging and inclusion. Belongingness constitutes a core dimension of social connectedness, reflecting subjective experiences of acceptance and valuation within groups (Costen et al., 2013; Plesko et al., 2021). This experience is closely linked to social identity affirmation and emotional attachment, enabling individuals to perceive themselves as recognized and cared for. Specifically, social support strengthens college students’ social connectedness by creating positive social experiences and interaction opportunities. On the other hand, social connectedness may also relieve the extent to which individuals experience ostracism. College students who lack stable social relations may be more likely to experience isolation, which in turn increases the risk of ostracism. Several theoretical studies also support that social connectedness is related to ostracism. According to social identity theory, individuals tend to define themselves by categorizing themselves into a certain social group, thereby fulfilling the basic psychological need for belongingness (Greenaway et al., 2015; Harwood, 2020). Social connectedness may strengthen this social identity through connections and interactions between individuals and group members, thus reducing the risk of ostracism. Combined with the above, it is reasonable to hypothesize that the relationship between social support and ostracism may be mediated by social connectedness. Specifically, individuals with adequate social support are more likely to establish more stable social connectedness, which provides them with emotional support and a sense of security, mitigating the risk of ostracism. Therefore, this study proposes H2: Social connectedness mediates the relationship between social support and ostracism.

### 1.3. The Moderating Role of Flourishing

Flourishing refers to an individual’s intrinsic positive psychological state that emphasizes optimal human functioning, focusing on fostering personal strengths and establishing positive social relationships (Diener et al., 2010; Keyes, 2002). This psychological state can affect the patterns and outcomes of individuals’ social interactions when they encounter life challenges. Previous empirical studies have consistently demonstrated significant associations between flourishing and both social support and social connectedness (Eraslan-Capan, 2016; Yildirim & Green, 2024). Specifically, individuals’ level of flourishing may significantly influence both the perceived intensity of social support and the degree of social connectedness with others. These findings suggest that flourishing may not only function as an independent protective resource but also interact with social support to jointly influence social connectedness. According to the protective–protective model, one protective factor can enhance the effect of another on outcome variables (Evans et al., 2010; Hollister-Wagner et al., 2001). In social contexts, flourishing and social support operate as synergistic protective factors, enhancing both the perception and utilization of external support while facilitating higher-quality social connectedness. This mechanism aligns with Fredrickson’s broaden-and-build theory of positive emotions, which posits that positive psychological states expand cognitive and attentional capacities, promoting open and inclusive interpretations of social environments (Fredrickson, 2001). Individuals with high flourishing demonstrate enhanced sensitivity to benevolent intentions in social interactions, thereby enriching their interpersonal networks. Conversely, low flourishing constrains cognitive-affective processing, predisposing individuals to skeptical appraisals and impairing the recognition of available support. Even amidst abundant social resources, such limitations hinder the formation and maintenance of social connectedness. Therefore, this study proposes H3: Flourishing may serve as a moderator between social support and social connectedness. Specifically, individuals with greater flourishing show a stronger association between social support and social connectedness.

### 1.4. The Present Study

**Figure 1:**
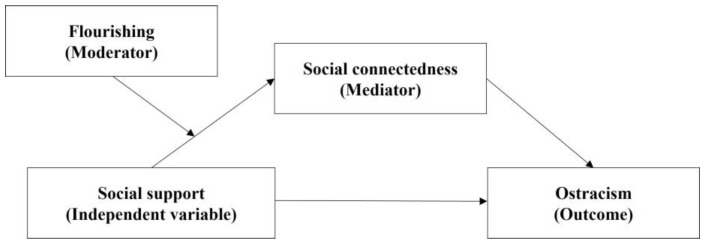
The proposed moderated mediation model.

This study aims to explore the underlying mechanisms between social support and ostracism among college students, as well as the mediating role of social connectedness and the moderating role of flourishing. Based on previous studies, we proposed the following hypotheses: (1) social support is significantly and negatively associated with ostracism; (2) social connectedness mediates the relationship between social support and ostracism; and (3) flourishing serves as a moderator between social support and social connectedness. The conceptual model is shown in Figure 1.

## 2. Materials and Methods

### 2.1. Participants

Data were collected from two universities in Heilongjiang Province, China, during May to June 2025 using a convenience sampling method. Questionnaires were administered during class breaks following ethical protocols: researchers explained the study’s purpose, procedures, and voluntary participation with unconditional withdrawal rights. After obtaining informed consent, 790 questionnaires were distributed. After excluding invalid responses, 723 valid samples were retained (91.52% validity rate). The sample comprised 434 females and 289 males (mean age = 19.39, SD = 0.91), including 482 freshmen, 184 sophomores, and 57 juniors. Furthermore, empirical research employing questionnaire methodologies generally recommends a minimum sample size of 10 times the number of measurement items (Nunnally, 1978). The sample size collected in this study satisfies this criterion.

### 2.2. Measures

#### 2.2.1. The Multidimensional Scale of Perceived Social Support

This scale was developed by (Zimet et al., 1988). This study used the Chinese version of the scale for measurement (Wang et al., 1999). This scale comprises twelve items on a 7-point Likert scale from 1 (strongly disagree) to 7 (strongly agree). Higher mean scores indicate higher levels of perceived social support. The Cronbach’s alpha coefficient for this scale in this study was 0.96.

#### 2.2.2. The Ostracism Experience Scale for Adolescents

This scale was developed by (Gilman et al., 2013). This study used the Chinese version of the scale for measurement (Zhang et al., 2018). The scale consists of eleven items on a 5-point Likert scale from 1 (never) to 5 (always). Higher mean scores indicate more ostracism experienced by participants in daily life. The internal consistency for this scale in the current study was 0.89.

#### 2.2.3. The Social Connectedness Scale

This scale was developed by (Lee et al., 2001). This study used the Chinese version of the scale for measurement (Fan et al., 2015). The scale consists of twenty items on a 6-point Likert scale from 1 (strongly disagree) to 6 (strongly agree). Higher mean scores indicate higher feelings of social connectedness. In this study, the Cronbach’s alpha coefficient for this scale was 0.90.

#### 2.2.4. The Flourishing Scale

This scale was developed by (Diener et al., 2010). This study used the Chinese version of the scale for measurement (Tang et al., 2016). This scale comprises eight items on a 7-point Likert scale from 1 (strongly disagree) to 7 (strongly agree). Higher mean scores indicate greater flourishing of participants. The scale demonstrated high internal consistency (i.e., 0.96) in this study.

### 2.3. Data Analysis

The study was statistically analyzed using SPSS 26.0 to calculate descriptive statistics and correlation coefficients for all variables. The mediating effect of social connectedness was further tested with the SPSS PROCESS macro (Model 4). Additionally, the PROCESS macro (Model 7) was employed to test the moderating role of flourishing between social support and social connectedness. Using the bias-corrected bootstrap method with 5000 resamples, we obtained 95% confidence intervals (CIs) for the parameter estimates (Hayes, 2013).

## 3. Results

### 3.1. Common Method Bias Test and Multicollinearity Diagnosis

Our data were collected using the questionnaire method, which may lead to common method bias (Podsakoff et al., 2003). To mitigate common method bias, anonymous reporting was employed during data collection, eliminating the need for identifiable information. Statistically, Harman’s single-factor test revealed eight factors with eigenvalues exceeding 1 in unrotated factor analysis, with the first factor accounting for less than 40% of total variance, indicating no severe common method variance. Additionally, variance inflation factors (VIFs) for all variables were below 5, confirming absence of multicollinearity.

### 3.2. Correlation Analysis

As shown in Table 1, significant positive correlations were found among college students’ social support, flourishing, and social connectedness. Furthermore, ostracism demonstrated significant negative associations with social support, social connectedness, and flourishing.

### 3.3. Testing for Mediation Effect

To examine the mediating role of social connectedness between social support and ostracism, mediation analysis was conducted using Model 4 from Hayes’ PROCESS macro for SPSS (Hayes, 2013), with gender included as a covariate (Table 2). The first step showed that social support significantly positively predicted social connectedness (*β* = 0.54, *p* < 0.001); the second step indicated that social support significantly negatively predicted ostracism (*β* = −0.40, *p* < 0.001); the third step showed that social connectedness significantly negatively predicted ostracism (*β* = −0.53, *p* < 0.001), and that social support still significantly negatively predicted ostracism (*β* = −0.11, *p* < 0.01). Therefore, social connectedness mediated the relationship between social support and ostracism among college students.

### 3.4. Testing the Moderated Mediation Effect

To test the moderating effect of flourishing on the relationship between social support and social connectedness, Model 7 from Hayes’ PROCESS macro was employed (Hayes, 2013), controlling for gender (Table 3). The interaction between social support and flourishing had a significant predictive effect on social connectedness (*β* = 0.15, *p* < 0.001). Therefore, the moderating effect of flourishing on the first path of the mediation model was significant.

**Figure 2:**
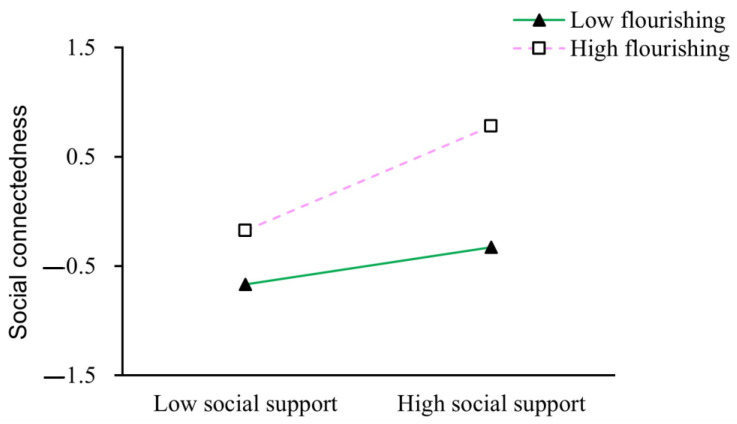
Flourishing moderates the relationship between social support and social connectedness.

To further examine the moderating effect of flourishing on the relationship between social support and social connectedness, participants were divided into two groups based on flourishing scores: high flourishing group (*M* + 1*SD*) and low flourishing group (*M* − 1*SD*). The predictive effect of social support on social connectedness was subsequently tested in two groups. As shown in Figure 2, social support significantly positively predicted social connectedness among college students with higher flourishing (*β*_simple slope_ = 0.48, *p* < 0.001). In contrast, for college students with lower flourishing, social support also showed a significant positive predictive effect (*β*_simple slope_ = 0.17, *p* < 0.001). However, its predictive effect was weaker. This indicates that the positive effect of social support on social connectedness is more pronounced among individuals with higher levels of flourishing, whereas this effect is attenuated among those with lower flourishing. This differential pattern underscores the critical role of high flourishing in amplifying the pathway of social support to social connectedness.

## 4. Discussion

This study revealed the negative relationship between social support and ostracism in college students, and examined the mediating role of social connectedness and the moderating effect of flourishing. The findings may lay a theoretical foundation for future research on ostracism, and are of great significance for enhancing social support, reducing ostracism, and improving social connectedness and flourishing among college students.

### 4.1. Social Support and Ostracism

The results indicated that social support was significantly and negatively associated with ostracism. College students with higher perceived social support tend to exhibit lower ostracism, supporting H1. This finding is consistent with a previous study, which highlights the role of social support in improving psychological well-being (Yao et al., 2015). College students with greater social support are often accompanied by lower levels of ostracism, which aligns with the buffer theory of social support. This theory suggests that individuals require and benefit from the support and assistance of others, which serves as a buffer against life pressure (Alloway & Bebbington, 1987; Cohen & Wills, 1985). As integral members of society, college students exhibit fundamental needs for recognition, support, and respect within their social groups. When experiencing social support and encouragement from others, these positive interpersonal resources can effectively alleviate the risk of ostracism, thereby fulfilling their innate psychological needs for social belonging and support. Furthermore, the buffering effect of social support on ostracism may be influenced by cultural value orientations, such as within Chinese cultural contexts, where collective goals and interpersonal harmony are emphasized (Triandis et al., 1988). Consequently, the ostracism of college students may be relieved when they receive social support. This finding emphasizes the importance of establishing and maintaining positive social relationships in reducing ostracism and promoting the psychological well-being of college students.

### 4.2. The Mediating Role of Social Connectedness

This study reveals the mediating role of social connectedness between social support and ostracism; social support reduces the sense of ostracism through enhancing college students’ social connectedness, supporting H2. This research not only extends existing theoretical frameworks but also provides novel empirical perspectives. Specifically, it examines the mediating role of social connectedness in linking social support with ostracism, providing new evidence for identifying social influencing factors that enhance college students’ psychological well-being.

Our findings support the important role of social support in enhancing social connectedness. Individuals who receive more social support tend to experience higher levels of belonging, membership, and identification with the community (Pang, 2020). This positive affective experience reinforces mutual trust among social members, thereby consolidating social connectedness and enhancing individuals’ motivation to value and maintain their social network relationships. This finding underscores the importance of strengthening social support networks to foster healthy interpersonal development and prevent potential negative psychological consequences. It should be noted that the study sample consisted of Chinese college students. Influenced by collectivist cultural norms, individuals emphasize interdependence and maintaining relationships (Gabrenya & Hwang, 1996; Lun & Bond, 2006). Consequently, social support is perceived not merely as emotional or instrumental resources, but as a crucial foundation for social connectedness and identity validation. This cultural context may amplify the role of social support in facilitating social connectedness. The results demonstrate a significant negative correlation between social connectedness and ostracism. According to social identity theory, human beings are born with basic needs for recognition and acceptance, and when individuals lack social connectedness, they may be more likely to experience ostracism (Greenaway et al., 2015; Harwood, 2020). Individuals with high social connectedness can develop greater psychological resilience against the adverse effects of ostracism, owing to robust social networks that provide both emotional support and a sense of identity.

### 4.3. The Moderating Role of Flourishing

Consistent with our expectations, flourishing moderated the relationship between social support and ostracism, supporting H3. Flourishing not only positively predicted social connectedness but also moderated the effect of social support on social connectedness. Specifically, the association between social support and social connectedness was stronger for college students with high flourishing. This finding supports the protective–protective model, in which one factor can reinforce the effect of another on an outcome variable (Evans et al., 2010; Hollister-Wagner et al., 2001). According to this model, within the challenging university environment, students with higher levels of flourishing tend to cognitively appraise their surroundings as opportunity-rich contexts (Datu, 2018). They are more willing to proactively seek and accept help from others, as well as to offer help to others, thus strengthening social connectedness. In contrast, college students with lower levels of flourishing may be more likely to encounter stress in the face of challenges. They may also be relatively less likely to perceive and utilize social support, thus reducing opportunities to build strong bonds with others. This finding also fits with the broaden-and-build theory of positive emotions (Fredrickson, 2001). This theory suggests that positive emotions and traits expand the cognitive and behavioral scope of individuals, contributing to more social engagement and active interaction. Individuals’ perceptions and utilization of social relationship quality are significantly influenced by their psychological states (Zheng & Gunasekara, 2022). Positive psychological states (e.g., mindfulness) can activate individuals to seek and utilize social support, which in turn facilitates the formation and development of social connectedness.

## 5. Practical Implications

This study provides significant implications for mitigating ostracism and promoting positive psychological adaptation among college students. First, as social support constitutes a key resource against ostracism, teachers in college counseling centers should develop evidence-based enhancement strategies. These may include implementing peer mentoring systems and psychological support groups to provide emotional support and experiential sharing, enabling students to receive timely assistance during academic, interpersonal, or life challenges. Second, the findings reveal that social connectedness mediates the social support–ostracism relationship, while flourishing moderates this pathway. This underscores the importance of strengthening both social connectedness and flourishing. Specifically, social connectedness fosters collective identity and self-worth through group activities, thereby reducing ostracism experiences. Flourishing, as a positive psychological resource, enhances perceived social support and maintains adaptive coping during adversity. Consequently, college psychological counselors should employ positive psychology interventions to cultivate flourishing and encourage participation in student organizations to expand social networks. These multi-level mechanisms, including social support, social connectedness, and flourishing, collectively attenuate the detrimental effects of ostracism.

## 6. Limitations and Future Directions

This study utilized a cross-sectional design and cannot establish causal inferences between variables. Although the findings align with theoretical hypotheses, reverse causality remains plausible (e.g., ostracism may reduce social support). Future research should employ cross-lagged panel analyses or intensive longitudinal designs to better assess causal relationships and temporal dynamics. Second, the lack of control over the sequence in questionnaire administration may introduce order effects, potentially confounding results. Third, while focusing on comprehensive social support, this study did not examine differential predictive effects of distinct support sources (e.g., familial vs. peer support) on social connectedness or ostracism. Although demographic variables (gender, age, grade) were measured, critical factors like academic discipline and socioeconomic status were unmeasured, limiting interpretability and generalizability. Future research should compare the unique effects of support sources and incorporate additional demographic covariates. Finally, the sample of Chinese college students may reflect culturally specific mechanisms underpinning social support and connectedness within collectivist contexts. Cross-cultural comparisons are needed to validate the model’s applicability across individualistic and collectivist societies.

## 7. Conclusions

This study provides novel empirical evidence regarding how social support influences ostracism among college students and offers theoretical innovations. Unlike previous research focusing primarily on direct effects, this study introduces social connectedness as a mediating variable, elucidating the underlying mechanism between social support and ostracism. Furthermore, by incorporating flourishing as a moderator, we highlight the critical role of individual psychological resources in the formation of social connectedness. Consequently, the proposed moderated mediation model addresses limitations in existing research on related mechanisms and provides a theoretical framework for future interventions. Practically, these findings suggest that college administrators can mitigate the adverse effects of ostracism by enhancing social support, fostering social connectedness, and leveraging the protective function of flourishing. However, the exclusive reliance on Chinese student samples limits the generalizability of findings. Future cross-cultural studies should validate the applicability of this model across diverse cultural contexts to enhance its explanatory power and transcultural value.

## Figures and Tables

**Table 1:** Descriptive statistics and correlation coefficients for each variable.

Variable	*M*	*SD*	1	2	3	4
1. Social support	5.17	1.12	1			
2. Ostracism	2.37	0.66	−0.40 **	1		
3. Social connectedness	3.91	0.66	0.54 **	−0.59 **	1	
4. Flourishing	5.05	1.10	0.63 **	−0.43 **	0.60 **	1

Notes: *M* is mean value; *SD* is standard deviation. ** *p* < 0.01.

**Table 2:** Testing for mediation effect.

Dependent Variable	Independent Variable	*R* ^2^	*F*	*β*	*SE*	95%CI	*t*
Social connectedness	Gender	0.30	153.26 ***	−0.05	0.06	[−0.23, 0.02]	−1.61
	Social support			0.54	0.03	[0.48, 0.61]	17.45 ***
Ostracism	Gender	0.16	68.63 ***	0.02	0.07	[−0.11, 0.17]	0.44
	Social support			−0.40	0.03	[−0.47, −0.33]	−11.71 ***
Ostracism	Gender	0.36	133.42 ***	−0.01	0.06	[−0.14, 0.10]	−0.38
	Social support			−0.11	0.04	[−0.18, −0.04]	−3.11 **
	Social connectedness			−0.53	0.04	[−0.60, −0.46]	−14.87 ***

Notes: ** *p* < 0.01, *** *p* < 0.001.

**Table 3:** Testing the moderated mediation effect.

Dependent Variable	Independent Variable	*R* ^2^	*F*	*β*	*SE*	95%CI	*t*
Social connectedness	Gender	0.44	142.81 ***	−0.08	0.06	[−0.18, 0.04]	−1.34
	Social support			0.32	0.04	[0.25, 0.40]	8.87 ***
	Flourishing			0.40	0.04	[0.33, 0.47]	11.09 ***
	Social support flourishing			0.15	0.02	[0.11, 0.20]	6.93 ***

Notes: *** *p* < 0.001.

## Data Availability

The datasets generated and analyzed during the current study are available from the corresponding author upon reasonable request.

## References

[B1-behavsci-15-01198] Alloway R., Bebbington P. (1987). The buffer theory of social support—A review of the literature. Psychological Medicine.

[B2-behavsci-15-01198] Cohen S. (2004). Social relationships and health. American Psychologist.

[B3-behavsci-15-01198] Cohen S., Wills T. A. (1985). Stress, social support, and the buffering hypothesis. Psychological Bulletin.

[B4-behavsci-15-01198] Cornwell E. Y., Waite L. J. (2009). Social disconnectedness, perceived isolation, and health among older adults. Journal of Health and Social Behavior.

[B5-behavsci-15-01198] Costen W. M., Waller S. N., Wozencroft A. J. (2013). Mitigating race: Understanding the role of social connectedness and sense of belonging in African-American student retention in hospitality programs. Journal of Hospitality Leisure Sport & Tourism Education.

[B6-behavsci-15-01198] Datu J. A. D. (2018). Flourishing is associated with higher academic achievement and engagement in filipino undergraduate and high school students. Journal of Happiness Studies.

[B7-behavsci-15-01198] Diener E., Wirtz D., Tov W., Kim-Prieto C., Choi D.-W., Oishi S., Biswas-Diener R. (2010). New well-being measures: Short scales to assess flourishing and positive and negative feelings. Social Indicators Research.

[B8-behavsci-15-01198] Eraslan-Capan B. (2016). Social connectedness and flourishing: The mediating role of hopelessness. Universal Journal of Educational Research.

[B9-behavsci-15-01198] Evans W. P., Marsh S. C., Weigel D. J. (2010). Promoting adolescent sense of coherence: Testing models of risk, protection, and resiliency. Journal of Community & Applied Social Psychology.

[B10-behavsci-15-01198] Fan X.-L., Wei J., Zhang J.-F. (2015). On reliability and validity of social connectedness Scale-Revised in Chinese middle school students. Journal of Southwest China Normal University (Natural Science Edition).

[B11-behavsci-15-01198] Fredrickson B. L. (2001). The role of positive emotions in positive psychology: The broaden-and-build theory of positive emotions. American Psychologist.

[B12-behavsci-15-01198] Gabrenya W. K., Hwang K.-K. (1996). Chinese social interaction: Harmony and hierarchy on the good earth. The handbook of Chinese psychology.

[B13-behavsci-15-01198] Gençöz T., Özlale Y. (2004). Direct and indirect effects of social support on psychological well-being. Social Behavior and Personality.

[B14-behavsci-15-01198] Gilman R., Carter-Sowell A., DeWall C. N., Adams R. E., Carboni I. (2013). Validation of the ostracism experience scale for adolescents. Psychological Assessment.

[B15-behavsci-15-01198] Greenaway K. H., Haslam S. A., Cruwys T., Branscombe N. R., Ysseldyk R. (2015). From “We” to “Me”: Group identification enhances perceived personal control with consequences for health and well-being. Journal of Personality and Social Psychology.

[B16-behavsci-15-01198] Harandi T. F., Taghinasab M. M., Nayeri T. D. (2017). The correlation of social support with mental health: A meta-analysis. Electronic Physician.

[B17-behavsci-15-01198] Harwood J. (2020). Social identity theory. The international encyclopedia of media psychology.

[B18-behavsci-15-01198] Hayes A. (2013). Introduction to mediation, moderation, and conditional process analysis: A regression-based approach. Methodology.

[B19-behavsci-15-01198] Hollister-Wagner G. H., Foshee V. A., Jackson C. (2001). Adolescent aggression: Models of resiliency. Journal of Applied Social Psychology.

[B20-behavsci-15-01198] Hu S., Cai D., Zhang X. C., Margraf J. (2022). Relationship between social support and positive mental health: A three-wave longitudinal study on college students. Current Psychology.

[B21-behavsci-15-01198] Keyes C. L. M. (2002). The mental health continuum: From languishing to flourishing in life. Journal of Health and Social Behavior.

[B22-behavsci-15-01198] Lavasani M. G., Borhanzadeh S., Afzali L., Hejazi E. (2011). The relationship between perceived parenting styles, social support with psychological well-being. Procedia—Social and Behavioral Sciences.

[B23-behavsci-15-01198] Lee R. M., Draper M., Lee S. (2001). Social connectedness, dysfunctional interpersonal behaviors, and psychological distress: Testing a mediator model. Journal of Counseling Psychology.

[B24-behavsci-15-01198] Li S., Zhao F., Yu G. (2021). Social exclusion and depression among college students: A moderated mediation model of psychological capital and implicit theories. Current Psychology.

[B25-behavsci-15-01198] Lu L., Chen C. S. (1996). Correlates of coping behaviours: Internal and external resources. Counselling Psychology Quarterly.

[B26-behavsci-15-01198] Lun V. M.-C., Bond M. H. (2006). Achieving relationship harmony in groups and its consequence for group performance. Asian Journal of Social Psychology.

[B27-behavsci-15-01198] Morese R., Lamm C., Bosco F. M., Valentini M. C., Silani G. (2019). Social support modulates the neural correlates underlying social exclusion. Social Cognitive and Affective Neuroscience.

[B28-behavsci-15-01198] Niu G.-F., Shi X.-H., Yao L.-S., Yang W.-C., Jin S.-Y., Xu L. (2023). Social exclusion and depression among undergraduate students: The mediating roles of rejection sensitivity and social self-efficacy. Current Psychology.

[B29-behavsci-15-01198] Nunnally J. C. (1978). Psychometric theory.

[B30-behavsci-15-01198] Pang H. (2020). Examining associations between university students’ mobile social media use, online self-presentation, social support and sense of belonging. Aslib Journal of Information Management.

[B31-behavsci-15-01198] Plesko C. M., Yu Z., Tobin K., Gross D. (2021). Social connectedness among parents raising children in low-income communities: An integrative review. Research in Nursing & Health.

[B32-behavsci-15-01198] Podsakoff P. M., MacKenzie S. B., Lee J.-Y., Podsakoff N. P. (2003). Common method biases in behavioral research: A critical review of the literature and recommended remedies. Journal of Applied Psychology.

[B33-behavsci-15-01198] Schwarzer R., Knoll N. (2007). Functional roles of social support within the stress and coping process: A theoretical and empirical overview. International Journal of Psychology.

[B34-behavsci-15-01198] Scott K. L., Zagenczyk T. J., Schippers M., Purvis R. L., Cruz K. S. (2014). Co-worker exclusion and employee outcomes: An investigation of the moderating roles of perceived organizational and social support. Journal of Management Studies.

[B35-behavsci-15-01198] Tang X., Duan W., Wang Z., Liu T. (2016). Psychometric evaluation of the simplified Chinese version of flourishing scale. Research on Social Work Practice.

[B36-behavsci-15-01198] Teng F., Chen Z. (2012). Does social support reduce distress caused by ostracism? It depends on the level of one’s self-esteem. Journal of Experimental Social Psychology.

[B37-behavsci-15-01198] Triandis H. C., Bontempo R., Villareal M. J., Asai M., Lucca N. (1988). Individualism and collectivism—Cross-cultural perspectives on self ingroup relationships. Journal of Personality and Social Psychology.

[B38-behavsci-15-01198] Wang X., Wang X., Ma H. (1999). Rating scales for mental health.

[B39-behavsci-15-01198] Wickramaratne P. J., Yangchen T., Lepow L., Patra B. G., Glicksburg B., Talati A., Adekkanattu P., Ryu E., Biernacka J. M., Charney A., Mann J. J., Pathak J., Olfson M., Weissman M. M. (2022). Social connectedness as a determinant of mental health: A scoping review. PLoS ONE.

[B40-behavsci-15-01198] Williams K. D. (2002). Ostracism: The power of silence.

[B41-behavsci-15-01198] Williams K. D. (2007a). Ostracism. Annual Review of Psychology.

[B42-behavsci-15-01198] Williams K. D. (2007b). Ostracism: The kiss of social death. Social and Personality Psychology Compass.

[B43-behavsci-15-01198] Williams K. D., Zanna M. P. (2009). Ostracism: A temporal need-threat model. Advances in experimental social psychology.

[B44-behavsci-15-01198] Yao T., Zheng Q. Y., Fan X. C. (2015). The impact of online social support on patients’ quality of life and the moderating role of social exclusion. Journal of Service Research.

[B45-behavsci-15-01198] Yildirim M., Green Z. A. (2024). Social support and resilience mediate the relationship of stress with satisfaction with life and flourishing of youth. British Journal of Guidance & Counselling.

[B46-behavsci-15-01198] Zhang D., Huang L., Dong Y. (2018). Reliability and validity of the ostracism experience scale for adolescents in Chinese adolescence [青少年社会排斥量表在我国青少年中的信效度检验]. Chinese Journal of Clinical Psychology.

[B47-behavsci-15-01198] Zheng C., Gunasekara A. (2022). Sustaining workforce engagement: From mindfulness to psychological flourishing. Sustainability.

[B48-behavsci-15-01198] Zimet G. D., Dahlem N. W., Zimet S. G., Farley G. K. (1988). The multidimensional scale of perceived social support. Journal of Personality Assessment.

